# Distance learning ects and flipped classroom in the anatomy learning: comparative study of the use of augmented reality, video and notes

**DOI:** 10.1186/s12909-016-0757-3

**Published:** 2016-09-01

**Authors:** Javier Ferrer-Torregrosa, Miguel Ángel Jiménez-Rodríguez, Javier Torralba-Estelles, Fernanda Garzón-Farinós, Marcelo Pérez-Bermejo, Nadia Fernández-Ehrling

**Affiliations:** 1 Department of Podiatry, School of Physiotherapy and Podiatry, Catholic University of Valencia San Vicente Martir, C/ Ramiro de Maeztu 14, Torrente, 46900 Spain; 2 Didactics and Educational Innovation, School of Psychology, Teaching and Educational Sciences, Catholic University of Valencia San Vicente Martir, Valencia, Spain; 3 Faculty of Nursing, Catholic University of Valencia San Vicente Martir, Valencia, Spain; 4 Doctoral School Catholic University of Valencia San Vicente Martir, Valencia, Spain

**Keywords:** ECTS, Flipped classroom, Metacognition, Anatomy, Augmented reality, Autonomous learning

## Abstract

**Background:**

The establishment of the ECTS (European Credit Transfer System) is one of the pillars of the European Space of Higher Education. This way of accounting for the time spent in training has two essential parts, classroom teaching (work with the professor) and distance learning (work without the professor, whether in an individual or collective way). Much has been published on the distance learning part, but less on the classroom teaching section. In this work, the authors investigate didactic strategies and associated aids for distance learning work in a concept based on flipped classroom where transmitting information is carried out with aids that the professor prepares, so that the student works in an independent way before the classes, thus being able to dedicate the classroom teaching time to more complex learning and being able to count on the professor’s help.

**Methods:**

Three teaching aids applied to the study of anatomy have been compared: Notes with images, videos, and augmented reality. Four dimensions have been compared: the time spent, the acquired learnings, the metacognitive perception, and the prospects of the use of augmented reality for study.

**Results:**

The results show the effectiveness, in all aspects, of augmented reality when compared with the rest of aids.

The questionnaire assessed the acquired knowledge through a course exam, where 5.60 points were obtained for the notes group, 6.54 for the video group, and 7.19 for the augmented reality group. That is 0.94 more points for the video group compared with the notes and 1.59 more points for the augmented reality group compared with the notes group.

**Conclusions:**

This research demonstrates that, although technology has not been sufficiently developed for education, it is expected that it can be improved in both the autonomous work of the student and the academic training of health science students and that we can teach how to learn. Moreover, one can see how the grades of the students who studied with augmented reality are more grouped and that there is less dispersion in the marks compared with other materials.

## Background

One of the essential keys of the European Higher Education Area (EHEA) was the establishment of the European credits or ECTS (European Credit Transfer System) as a common measurement unit. Then, to complete the information and facilitate mobility and employment prospects of EU citizens, the Diploma Supplement was also established.

Accounting for credits based on the student’s learning time is a strategic option of very significant practical and theoretic impact. Furthermore, going from teaching to learning as the central point is an essential shift in the paradigm.

The fact is that we have a long way to go before ECTS is associated with the time spent by the student and it rather looks like a measurement linked to the teacher’s lecture hours: ten lecture hours one credit [1].

Clearly, if the real development of the ECTS is this, and not only in the Spain, it is because there are many practical difficulties. Some of them are to find the time students need for the accomplishment of the proposed tasks, the appraisal system still based on official call exams, which put off center the value of learning as opposed to the successful completion of tests, the difficulty of generating spaces for different types of grouping (active mentoring, seminaries, work groups,…) where classroom presence is based on the student and consequently also the difficulty of accounting for the professor’s time,… in the end, ten lecture hours and the rest “autonomous learning”, without the characteristics and integration of such learning being clear in the results of learning.

In this study, several questions have been addressed. The first was to focus the student’s autonomous learning on the specific schedule and the management of the professor as a basis for more complex learning, which can be carried out later in the classroom. We followed, therefore, the flipped classroom proposal [2].

The pedagogical approach of the convergence process is based on a basic principle: the centrality of learning. This transformation means myriad changes, starting with the change of mentality and even the deconstruction of our professors’ teaching models. The study targets two lines of work:

- To regard autonomous learning as a prior and central factor for learning and real implementation of the ECTS
- The role of didactic aids that become resources able to generate high level knowledge and to make autonomous learning easier. Along these lines, new information and communication technologies have an important role to play.

Furthermore, we wanted to find out how to optimize these learnings with the help of the best didactic aids. Logically, it depends on the subject to be learnt, in this case Anatomy, specifically the extrinsic muscles of the foot.

Opting for Augmented Reality (AR) is justified by the evidence that, thanks to technological progress, it is ever more possible to achieve 3D models for Anatomy study [3, 4]. Nowadays, students are used to handling technologies like Internet, 3D video games, mobile phones, MP3 players, and other technological devices. We need to change didactic methods, and particularly didactic aids, in order to encourage students to use the abilities and intelligence they usually develop for studying.

Under these conditions, AR (a version of virtual reality (VR) [5]) appears to be a smart technology that promises to offer the tools required to create attractive and motivating content [6]. Several researchers have experimented with the use of AR in teaching different parts of anatomy [7–11]. Other studies have investigated how important these tools are in digital literacy in the environment or context of the current knowledge society, defining the limits of rationality of virtual worlds, evaluating the meaning of AR [12]. It is quite clear that more and more schools, for instance in the U.S.A., are using AR for teaching [13].

Our research team decided to contact the LabHuman company, Polytechnic University of Valencia, which has vast experience [14–16] in the world of VR and AR. They are experienced in performing Magic book [17, 18] which is one of the most renowned AR educational applications.

### Hypothesis and aims

This research work aims to verify three hypotheses connected with each other, which are the following:

- Augmented reality provides a higher degree of learning than traditional videos and notes for anatomy study when they are used independently by the student in the method called flipped classroom.
- Autonomous learning with AR aids is more highly valued in the metacognitive perception of students than the use of videos or notes on the same subject (extrinsic muscles of the foot).
- The expectation of success in future learning of students who use AR aids is significantly higher than those who think that these aids will not contribute to such success.

The research work has been carried out covering the following aims:

- Development of didactic aids for autonomous learning
- Traditional notes with illustrations
- Voice-over narration video
- AR book
- To verify and analyze the conditions of time spent on each of the aids to better fit the workload in the distance learning ECTS
- To assess the results of the student’s autonomous learning with each of the aids through a common test for objective assessment
- To know the assessment that students make from a metacognitive perspective of each of the three types of given didactic aids
- To know the expectation of success in anatomy learning thanks to the use of AR from, mainly, the students who have experienced it.

## Methods

This project was developed during the first four-month period of the 2013/2014 academic year at the Catholic University of Valencia “San Vicente Martir” in the Area of Health Sciences, in the degrees of Medicine, Physiotherapy, and Podiatry, in the Anatomy taught in the first year and the topic chosen was the extrinsic muscles of the foot.

### Design

The research has a quasi-experimental transversal comparison of equivalent groups design.

### Participants and distribution of the sample

The selection of participants was random and included all the students who had attended lessons the day of the proposal and who voluntarily wanted to participate.

We divided each classroom into three groups, one for each didactic aid.

- Didactic aid 1 consisted of the professor’s notes on the subject of the extrinsic muscles of the foot with the help of anatomical atlas images. This is the typical didactic aid that students are usually presented with.
- Didactic aid 2 consisted of the same professor’s notes and a video on an anatomical corpse showing each of the structures of the notes, in addition to voice-over narration. The duration of this video is 22:58 min and can be watched on https://www.youtube.com/watch?v=Lg4qSwtnqDg&feature=youtu.be
- Didactic aids 3 consisted of the professor’s notes and AR software. These professor’s notes had built-in marks to be able to visualize three-dimensional objects.

One hundred seventy-one students were included: 78 from the Medicine Degree (24 Notes, 22 Video, 24 AR), 48 from the Physiotherapy Degree (22 Notes, 13 Video, 22 AR), and 45 from the Podiatry Degree (14 Notes, 17 Video, 14 AR) of whom 80 (46.8 %) were men and 91 were women (53.2 %) with an average age of 19.77 years (Desv tip 4,088).

Each student had to make a work manual, with five activities, using supporting material, such as the notes with images, the narrated video, and the AR book, respectively.

### Measuring tools

We used three tools:

1. A questionnaire in which they had to record the number of minutes used.
2. An appraisal exercise about the learning sought after in autonomous learning with aids.
3. A previously closed and validated questionnaire [19] that would allow measuring the metacognitive variables related to the use of each of the materials and the expectations in relation to learning in the use of the AR.

### Research development

Each of the three groups involved (Medicine, Podiatry, and Physiotherapy which are those that have the study of the chosen subject, i.e., extrinsic muscles of the foot within their curriculum) was subdivided in turn into three groups based on the type of aids they were going to work with. They were given a manual with five specific and common activities that would help them to assimilate the contents to be learned and they were also given a form in which to record the time spent on the preparation of these activities.

They were given a deadline of 14 days for the preparation and they were not advised that there would be an assessment test so as not to condition this process.

Once the deadline was up, the assessment test was carried out on the contents the students had prepared, which would be used as a starting point for the development of more complex elements (following the flipped classroom sequence) and a questionnaire to assess the metacognitive aspects and the expectations of learning (when AR was to be used in the future for anatomy study), respectively.

## Results

We will explain the results according to the measuring tool used.

### Analysis of the time spent

The average time spent based on the aids used was the following: Notes Group: 182.95 min, Video Group: 140.83 min, and AR Group: 189.35 min.

### Learning

Measuring of learning in each of the groups was performed with an objective test (exam) to assess the acquisition of Anatomy contents, in each of the groups. The highest possible score in this test is ten points.

After the calculations made on the means and the variances (ANOVA) we found very relevant data. The difference of means is significant (*p*-value < 0.000) between the results of the students who used AR when compared with those of using video and it is also significant between the use of video and notes.

**Fig. 1:**
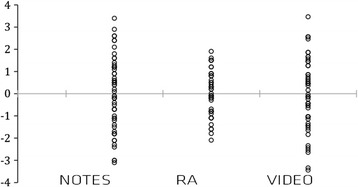
Remainders

The average mark obtained with AR (7.20 points) is significantly higher than that obtained with video (6.54 points), which in turn is significantly higher than that obtained with the notes (5.61 points). In addition, we have observed that the method used has a significant effect on dispersion, which is specified by the dispersion of scores being significantly lower with the AR method than with either of the other two methods. (See Fig. 1.)

### Metacognitive results on the study with different aids

*Metacognition is the way of learning how to reason about the reason itself, to apply the thought to the act of thinking, to learn to learn, it is improving the intellectual activities and tasks we carry out using reflection to guide them and to ensure a good execution.* (Yael Abramovicz Rosenblatt). There are many variables that could be analyzed to analyze such a complicated construct.

Our team obtained the results using the specifically validated questionnaire; we are going to address the perception students showed on the following aspects (see Table 1).

**Table 1:** Anova results Block I

			ANOVA of a factor				
			Addition of	gl	Mean	F	Sig.
Block I. Attention and motivation	1. - It helps me to fix my attention.	1	8.573	2	4.287	7.335	.001*
		2	98.176	168	.584		
	2. - It helps me to retain the contents.	1	3.377	2	1.688	2.920	3.057
		2	97.126	168	.578		
	3. - It motivates me to learn.	1	3.117	2	1.559	2.227	.111
		2	117.596	168	.700		
	4. - It makes possible studying in different ways avoiding in this way feeling frustration.	1	15.682	2	7.841	10.887	.000*
		2	120.996	168	.720		
	5. - It helps me to see/to imagine very clearly what I am being explained.	1	25.262	2	12.631	18,402	.000*
		2	115.311	168	.686		
	6. - It helps me to understand foot biomechanics.	1	5.361	2	2.681	3.748	.026*
		2	120.159	168	.715		
	7. - It helps me to understand the subject without excessive explanations from the professor.	1	2.166	2	1.083	2.102	.125
		2	86.582	168	.515		
	8. - It helps me to revise at home.	1	.621	2	.311	.483	.618
		2	108.057	168	.643		

1 (Intergroups); 2 (Intragroups)

*p-value > 0.05

Block I Attention and Motivation, Block II Work or autonomous learning, Block III Three-dimensional comprehension.

Regarding the use of aids as a help for holding attention, (Question 1 *p*-value > 0.001) 63.5 % of students preferred AR, as opposed to 33.9 % Notes Group and 51.7 % the Video Group,

Augmented reality helped to hold the student’s attention and together with the required learning curve, it correlates with the use of more study time and less distraction. Therefore, we can presume that when students use AR as a didactic resource they can improve their focus on the task of learning. It takes longer, but it results in an effective time for learning.

Another significant difference, question 4, (*p*-value > 0.000) is in the possibility noticed by students that AR provides a different option, they can, therefore, learn better than with the other two resources. The results are 75 % against 55 % video and 30.5 % notes with still images.

Questions 5 and 6 show an important and significant difference in both cases since the didactic aids with AR go a step further in explanations and three-dimensional comprehension of anatomy of the foot. So there is a *p*-value >0.000 and a *p*-value >0.026, respectively. Eighty-four point six per cent of the AR group consider that it helps them to see/imagine very well what is being explained to them as opposed to 66.7 % of the video group and 37 % of the notes group.

We can see this result regarding the following dimension, associated with the principles of the flipped classroom method, which is motivation for the autonomous learning.

In the first place, capacity of learning without the professor’s presence: *7. - It helps me to understand the subject without excessive explanations from the professor.* Question 7. In this case, the answers of the students, who were not used to this method, found that AR can also be helpful to them, or more useful than the notes or the video (63.5, 42.4, and 48.3 %).

When we analyze the results of the questions related to the block of autonomous learning, we can see statistically significant differences in the whole of Block II (see Table 2) and it becomes clear that in all the cases, use of AR surpasses the other two aids.

**Table 2:** Anova. Results Block II

			ANOVA of a factor				
			Addition of	gl	Mean	F	Sig.
Block II. Autonomous learning	9. - Active learning stimulates me.	1	4.633	2	2.316	3.297	.039*
		2	118.045	168	.703		
	10. - It strengthens my autonomous learning.	1	4.509	2	2.254	3.288	.040*
		2	115.175	168	.686		
	11. - It would allow me to repeat by myself, outside the university, the activities made in class.	1	6.200	2	3.100	4.401	.014*
		2	118.327	168	.704		

1 (Intergroups); 2 (Intragroups)

*p-value > 0.05

Students consider that AR helps and implements three-dimensional comprehension, which is an ideal complement for understanding complex subjects like Anatomy. Students value this resource positively in 52 % of cases as opposed to 26.6 % of the video group and 22 % of the notes group.

In all the items related to 3D understanding, where the perception of related elements and three-dimensional comprehension is essential, the difference between AR and the other two aids is significant, as Table 3 shows.

**Table 3:** Anova. Results Block III

			ANOVA of a factor				
			Addition of	gl	Mean	F	Sig.
Block III. Three-dimensional comprehension	12. - It is able to make me understand each movement perfectly.	1	5.011	2	2.505	5.752	.004*
		2	73.176	168	.436		
	13. - It has allowed me to know the parts of anatomy of the foot but not its global functioning.	1	5.040	2	2.520	4.656	.011*
		2	90.936	168	.541		
	14. - I have been able to understand anatomy.	1	5.830	2	2.915	6.110	.003*
		2	80.147	168	.477		
	15. - I have been able to understand the movements of each studied muscle.	1	4.761	2	2.380	5,067	.007*
		2	78.924	168	.470		
	15. - I have been able to visualize the movements of each studied muscle.	1	5.936	2	2.968	5.615	.004*
		2	88.812	168	.529		
	17. - I have been able to relate the different anatomical structures among themselves.	1	2.314	2	1.157	2.160	.119
		2	90.013	168	.536		

1 (Intergroups); 2 (Intragroups)

*p-value > 0.05

Just in the question “I have been able to relate the different anatomical structures to each other”, it can be seen that it is not significant, perhaps because all the structures in the AR Book were generated individually only in relation to bones in order to analyze their origin, insertion and function, and it has never been compared to adjacent structures.

### Expectations about learning with the use of AR as a didactic aid

Finally, we asked the students to imagine what consequences AR would have on their learning (see Table 4). Although the whole student sample answered regarding the expectations they had on the use of AR, and it was highly valued in all the groups, for the purpose of analysis we will take into consideration the opinions of those who did indeed use it in the experiment and therefore can give their opinion as they are fully informed, having tried it. The results are clearly positive: 76.9 % considered that AR would be effective for studying, 75 % felt that it can increase motivation and interest in the subject, and 67.3 % believed that if the professors used it their marks would improve.

**Table 4:** Frequencies (f) and Percentage (%) of answers about A.R.

		Notes		Video		A.R.	
		Yes	No	Yes	No	Yes	No
18. I consider using Augmented Reality in the Anatomy subject is efficient.	F	31	28	37	23	40	12
	%	52.50 %	47.50 %	61.70 %	38.3 %	76.90 %	23.1 %
19. I consider that the use of Augmented Reality can increase my interest for the subject.	F	38	21	39	21	39	13
	%	64.40 %	35.60 %	65.00 %	35.00 %	75.00 %	25.00 %
20. I believe I can get better marks if my professors use Augmented Reality as a didactic resource.	F	32	27	37	23	35	17
	%	54.20 %	45.8 %	61.7 %	38.3 %	67.30 %	32.70 %

## Discussion

The consolidation of EHEA has a long way to go because it is a change in the University’s theoretical paradigm and culture. One of the keys is in the effective implementation of the ECTS in both 40 % of classroom learning and 60 % of distance learning workload, which is often overlooked. It is essential to measure the students’ average work times as a whole because the personal variables produce very high dispersion. The records of the students’ experiences and trial and error are probably the way of adapting planning to reality, taking into account that the human variable of each group of students can provide approximate results.

The proposal of the method known as flipped classroom fits in well with the effective development of the ECTS. It seems that this approach of planning of learning time, whether classroom or distance learning, in which students are the focus, can give us a good perspective.

The didactic aids we make available to the students are the key. They do not need the professor’s presence and we expect to achieve significant learning. In order to analyze this problem (always in the context of Anatomy learning) three work hypotheses that we were able to verify were considered in the study.

The first hypothesis: AR *provides a higher learning degree than traditional video or notes for anatomy study when it is used independently by the student in the method called flipped classroom.*

This has been confirmed by the results of the students’ tests. Moreover, the group using AR as a resource obtained less dispersion in their grades, therefore there was a general improvement in learning.

We would probably obtain similar results if we applied virtual reality. The aids used by the student for the correct application of flipped classroom should make it possible to experiment, as many times as necessary, in order to acquire knowledge that will subsequently be ideal work material. On the basis of this acquired knowledge, the professor activates, in the classroom, processes of a high cognitive level in the students. Virtual reality, due to the strength of the sensorial experience it provides, is appropriate for this methodology.

The research results indicate that AR is an aid with which the student learns better and more, AR 7.20 points > Video (6.54 points) > Notes (5.61 points). Similar results were obtained in the study by Martin Gutierrez [20] in which the measurement of learning in that course was performed with an exam in which the highest possible score was 6 points. The participants obtain an average score of 5.71 points.

Regarding the second hypothesis: *Autonomous learning with AR aids is more highly valued in metacognitive perception by students than the use of video or notes on the same subject (extrinsic muscles of the foot)*.

We have also been able to see significant differences in all the items that comprised the construct that we referred to as metacognitive perception where the attention, retention, supply of alternatives, stimulus of motivation to achieve autonomous learning, and 3D comprehension were better valued when the students used AR.

The use of a novel tool may have had an influence on the motivation to study and the time spent in studying, both of which, obviously, bring about better results. Nonetheless, when AR is no longer novel, we believe it will still work as it will still provide resources to optimize the different types of intelligence of the students who will be able to apply spatial-visual as well as bodily-kinesthetic intelligence by this means, giving new opportunities to all the students and maintaining a lower dispersion, as occurred in the experiment.

Regarding the third hypothesis: *The expectation of success in the students’ future learning who experiment with AR aids is significantly higher than the expectation of those who think that these aids will not contribute to such a success.* We have also been able to confirm this. All the students, both those who did not use AR and those who did, have the feeling that AR can offer them good possibilities and in fact their expectation was significantly better after experiencing AR as a didactic aid.

We can also observe how technology can be the motor of educational change, especially if educational research is offering results. It must be framed in a good curricular design, we must understand why we use them and what we use them for. Dede points out that with the transformation of technologies, teachers are continuously developing new educational and learning methods [21].

It is true that the use of technology in teaching and learning entails some difficulty, which commercial companies endeavor to minimize by using ever more intuitive methods. At the beginning of our experiment, the students experienced some slight difficulties in handling RA aids. However, a few minutes sufficed to acquire the necessary handling skills. The introduction of AR in non-academic settings, especially in recreational elements, demonstrates that, progressively, students need less time to adapt to handling it.

This study confirms the premise introduced by Dede, since the use of technology as a didactic aid, specifically AR, brings about improvements and changes in teaching.

Martin Gutierrez [20] obtained a very good overall assessment of the course and most of the students considered it was very useful (67 %), very interesting (79 %), and they were satisfied with the technology and the method (83 %). All the participants (100 %) considered that the AR-Dehaes system was user-friendly and useful for the improvement of spatial abilities.

Comparing it with our study, they considered that it could increase their interest for the subject, 67.83 % (Question 23) of the students, regardless of the group to which they belonged, that is to say, similar to the 64.4 % in Martin-Gutierrez’s study.

There are studies that demonstrate the possibilities of AR in the same way as our study, some with simulations with AR together with exercises with feedback interfaces, proving the improvement of physical abilities [22].

We have attempted to show how very useful AR currently is on the basis of this research work, in the area of Anatomy knowledge. The use of visual aid is a very effective teaching method, and studies show an increase in memory retention when it is compared with the more traditional teaching methods. The student obtains a greater assimilation of information when using graphical information instead of data in texts. According to a study by Saettler et al., they found that students learned more, remembered more, and showed a greater interest when films were used in learning [23]. In addition, in 1984 it was proved that learning time is reduced when students are involved and become the active subject of their own education [24]. Comparing it with this research, we reject both theories since the students who used AR and video obtained better marks than the students who followed the traditional method and, furthermore, this study proves that AR technology can work independently, generating better academic results, although more time is spent on independent work, a fact caused by the learning curve that AR requires, among other variables already analyzed.

The didactic resource devised for this research made students use new technologies and they obtained an improvement that is proved statistically in different studies and in addition, as the study by Tallyn E, et al. [25] shows, we obtained a flexible interface which was readily accessible, rather like a paper book.

In this context, researches on the use of computer-aided tools show that they are very well accepted by students [26]. Other studies show that there is an increase in learning [27]. Already in 2001, St. Aubin made a study on simulation that was carried out in the Human Anatomy/Physiology classes as a voluntary and complementary part to the students’ training. They obtained positive results with respect to the simulation with virtual reality, improving the learning capacity of students [28]. In our research, 63.5 % of the students in the group that used AR are able to understand the movements of each muscle studied, 67.3 are able to relate the anatomical structures; by comparison, the notes group was only 40.7 and 49.2 %, respectively.

Augmented reality technology has, in addition, a positive impact on the students’ attention. In fact, it is one of the educational effects expected from new technologies from both 3D virtual worlds and AR [29]. Our study also demonstrates that it helped 63.5 % of the students of the AR group, 51.7 % of the video group, and only 33.9 % of the notes group to pay more attention.

From the data previously explained, in the part of the anatomy exam, we can see that students in the AR group improved their anatomy knowledge and obtained better marks, motivated by better comprehension of what they had to learn. At the same time, they recreated a more exact three-dimensional image of reality simultaneously understanding and visualizing more clearly muscle movements. We can conclude, in the same way as other studies [15] on spatial abilities, that the statistics show an improvement in students’ efficiency when using AR.

## Conclusions

This research demonstrates that, although technology has not been sufficiently developed for education, it is expected that it can be improved in both the autonomous work of the student and the academic training of health science students and that we can teach how to learn. Moreover, one can see how the grades of the students who studied with augmented reality are more grouped and that there is less dispersion in the marks compared with other materials.

## Abbreviations

AR: Augmented reality
ECTS: European Credit Transfer System
EHEA: European Higher Education Area
VR: Virtual reality
